# Genotypic characterization, antimicrobial susceptibility and virulence determinants of *Campylobacter jejuni* and *Campylobacter coli* isolated from pastured poultry farms

**DOI:** 10.3389/fmicb.2023.1271551

**Published:** 2023-11-09

**Authors:** Amal Awad, Hung-Yueh Yeh, Hazem Ramadan, Michael J. Rothrock

**Affiliations:** ^1^Department of Bacteriology, Mycology, and Immunology, Faculty of Veterinary Medicine, Mansoura University, Mansoura, Egypt; ^2^U.S. National Poultry Research Center, Agricultural Research Service, United States Department of Agriculture, Athens, GA, United States; ^3^Department of Hygiene and Zoonoses, Faculty of Veterinary Medicine, Mansoura University, Mansoura, Egypt

**Keywords:** *Campylobacter*, pasture-raised poultry, MLST, antimicrobial resistance, virulence

## Abstract

**Aim:**

*Campylobacter* is the leading bacterial pathogen that causes foodborne illnesses worldwide. Pasture farming is regarded as an important source of agricultural production for small farming communities. Consumer preference for pasture-raised animal products has increased; however, there is a paucity of information on the microbiological quality of pasture-raised poultry products. The purpose of this study was to explore genetic relatedness of thermophilic *Campylobacter* isolates, to assess antibiotic resistance phenotypically and genotypically, and to screen the presence of virulence determinants of *Campylobacter* isolates from pasture-raised poultry farms from southeastern United States.

**Methods:**

Ninety-seven *Campylobacter* isolates previously identified by Q7 BAX^®^ System Real-Time PCR were genotyped by multilocus sequence typing (MLST). *Campylobacter* isolates were then evaluated for their phenotypic antimicrobial susceptibility against nine antimicrobial agents using Sensititre plates. Additionally, *Campylobacter* isolates were tested for the presence of antimicrobial resistance-associated elements. Furthermore, *Campylobacter* isolates were screened for the presence of 13 genes encoding putative virulence factors by PCR. These included genes involved in motility (*flaA* and *flhA*), adhesion and colonization (*cadF*, *docC*, *racR*, and *virB11*), toxin production (*cdtA*, *cdtB*, *cdtC*, *wlaN*, and *ceuE*) and invasion (*ciaB* and *iamA*).

**Results:**

Among 97 *Campylobacter* isolates, *Campylobacter jejuni* (*n* = 79) and *Campylobacter coli* (*n* = 18) were identified. By MLST, *C. jejuni* isolates were assigned to seven clonal complexes. Among them, ST-353, ST-607 and ST-21 were the most common STs recognized. All *C. coli* (*n* = 18) isolates were included in CC-828. Interestingly, eight STs identified were not belonging any previous identified clonal complex. *Campylobacter* isolates displayed a high resistance rate against tetracycline (81.4%), while a low rate of resistance was observed against macrolides (azithromycin and erythromycin), quinolones and fluoroquinolones (nalidixic acid and ciprofloxacin), aminoglycosides (gentamicin), ketolide (telithromycin), amphenicol (florfenicol) and lincomycin (clindamycin). Thirteen isolates (13.54%) were pan-susceptible to all tested antibiotics, while nine isolates were multi-antimicrobial resistant (MAR; resist to three or more antimicrobial classes). Interestingly, there were no isolates resistant to all antimicrobial classes. Thr86Ile mutation was identified in all quinolones resistant strains. Erythromycin encoding gene (*ermB*) was identified in 75% of erythromycin resistant isolates. The A2075 mutation was detected in one erythromycin resistant strain, while A2074 could not be identified. The *tet*O gene was identified in 93.7% of tetracycline resistant isolates and six tetracycline susceptible isolates. In conclusion, the results of this study revealed that *Campylobacter* isolates from pasture-raised poultry farms showed the ST relatedness to *Campylobacter* isolates commonly associated with humans, indicating pasture-raised broiler flocks, similar to conventionally-reared broiler flocks, as a potential vector for antibiotic-resistant and pathogenic strains of thermophilic *Campylobacter* to humans.

## Introduction

Thermophilic *Campylobacter* spp., particularly *Campylobacter jejuni* and *Campylobacter coli*, have been established as leading causes of food-borne illnesses worldwide (Vetchapitak and Misawa, 2019; European Food Safety Authority, 2021; Sher et al., 2021). The U.S. Centers for Disease Control and Prevention (Centers for Disease Control and Prevention, 2022) estimated that 1.5 million U.S. residents are infected with *Campylobacter* each year. Most patients have acute, self-limiting gastroenteritis, but some may have severe and long-lasting illnesses, which require antibiotic treatment, particularly in immunocompromised patients (Ma et al., 2014). Additionally, the infection by *Campylobacter* may be associated with a number of complications such as polyarthralgia, Guillain-Barre syndrome (GBS), Miller Fisher syndrome and even death (Kaakoush et al., 2015).

Campylobacteriosis is transmitted by eating raw or undercooked poultry meat (Centers for Disease Control and Prevention, 2022). *Campylobacter* contaminates poultry meats prior to or during processing representing a potential health threat to consumers (Suzuki and Yamamoto, 2009). *Campylobacter* contamination in poultry farms could occur via feed, water, soil, contact animals, biosecurity threats, and vehicles (Ghareeb et al., 2013).

The survival and pathogenicity of *Campylobacter* species are all influenced by several virulence factors (Casabonne et al., 2016). Bacterial motility, adherence to the intestinal epithelial walls, colonization and cytotoxin production are the main bacterial virulence factors. Several genes related to *Campylobacter* virulence factors have recently been identified including adhesion and colonization (*flaA*, *flhA*, *cadF*, and *racR*), invasion-associated markers (*ciaB*, *iam*, and *virB11*), and ganglioside mimicry (*wlaN*) (Bolton, 2015).

There is a growing antibiotic resistance crisis in clinical medicine since antibiotics were historically used in food animal production either for treatment or for growth promotion, which led to human exposure and infection through a variety of pathways, including meat and poultry products (Price et al., 2007). Moreover, a significant portion of the antibiotics provided are not absorbed by the animals and are excreted in the urine and feces. In *Campylobacter* infections, antibiotic therapy is commonly required for immunocompromised patients and those with severe campylobacteriosis (Kaakoush et al., 2015). Generally, *Campylobacter* infections are treated with macrolides (erythromycin, clarithromycin, and azithromycin), although fluoroquinolones (ciprofloxacin) are the most effective drugs to treat diarrhea (Aarestrup et al., 2008). Additional alternative drugs for treatment are tetracycline, doxycycline, and chloramphenicol (Skirrow and Blaser, 2000).

Pastured poultry farms in the USA are considered an important source of animal production that may provide an important opportunity to strengthen rural communities (Conner et al., 2008). Consumer preference of free-range and pasture-raised animal products such as meat, milk, and eggs has grown (Stampa et al., 2020). Because there is a paucity of information on the quality of pasture-raised chickens, many customers feel that these products are of superior quality in contrast to conventionally-farmed chickens, due to their more natural growing conditions (Yeung and Morris, 2001). There is insufficient research on genotyping, presence of virulence determinants, and antibiotic resistance of *Campylobacter* isolates from pasture-raised poultry farms; therefore, the purpose of this study was to explore genetic relatedness of thermophilic *Campylobacter* isolated from pasture-raised poultry farms and the following processing operations of broiler carcasses, and to assess antibiotic resistance phenotypes and genotypes as well as to screen the presence of virulence determinants in the retrieved isolates.

## Materials and methods

The farm description, sample collection and processing, and *Campylobacter* isolation methods were previously described (Rothrock et al., 2016). Briefly, the samples were collected from feces, pasture soil, cecal content at processing, whole carcass rinsates and final whole carcass products. All samples were collected in the field and were brought back to the laboratories in a cooler packed in ice. The total amount of fecal and soil samples was at least 25 grams per sample. For homogenization, three grams (feces, cecal and soil samples) were diluted 1:3 in 10 mM phosphate buffered saline (PBS) in sterile filtered stomacher bags (Seward Laboratories, Inc., Bohemia, NY, United States). For rinsates, 100 mL of 10 mM PBS were added to each carcass within the storage bag, and the bags were vigorously shaken for 1 minute. The rinsates were collected into the sterile filtered stomacher bags (Seward Laboratories, Inc.). All samples were homogenized for 1 minute with a Stomacher^®^ 400 Blender (Seward Laboratories, Inc.), and these homogenates were used for the downstream *Campylobacter* isolation. A volume of 100 μL from the above homogenized suspension was plated onto Campy-Cefex agar (prepared in the laboratory; Stern et al., 1992). The plates were incubated at 42 ± 1°C for 36 to 48 h in a microaerobic condition (85% N_2_, 10% CO_2_ and 5% O_2_) (Hiett et al., 2008; Yeh et al., 2013). Presumptive *Campylobacter* colonies were selected and enumerated on Brucella agar supplemented with 10% lyzed horse blood for isolation (prepared in the laboratory; Stern et al., 1992). The plates were incubated as described above. Speciation of *Campylobacter* was carried out using a Q7 BAX Real-Time PCR system according to the manufacturer’s instructions as described previously (Yeh et al., 2022). An end-point multiplex PCR assays were also performed. The 16S rRNA primers specific to *Campylobacter* in the PCR assays generated amplicons both in *C. jejuni* and *C. coli* samples, verifying the isolates as *Campylobacter* (Linton et al., 1997). The PCR assays with *hipO* primers amplified a 323-bp product in the *C. jejuni* samples, but not in the *C. coli* samples, verifying the isolates as *C. jejuni* (Caner et al., 2008). The PCR with primers from the *ask* gene generated about a 550-bp gene fragment that identified the samples of *C. coli* (Linton et al., 1997). *Campylobacter* isolates were frozen at −80°C in Luria-Bertani broth with 20% glycerol until downstream analyses were performed.

### Bacterial cultures and genomic DNA isolation

*Campylobacter jejuni* (*n* = 79) and *C. coli* (*n* = 18) isolates from our stock in the U.S. National Poultry Research Center, Agricultural Research Service, U.S. Department of Agriculture, Athens, GA, United States were used in this study. Bacterial cultures were revived in Mueller-Hinton agar plates at 42°C for 48 h under the microaerobic condition as described as above.

DNA was extracted from pure bacterial cultures of 79 *C. jejuni* and 18 *C. coli* using the DNeasy Blood & Tissue Kit (Qiagen Inc., Germantown, MD, United States) in accordance with the manufacturer’s instructions. DNA concentrations were measured spectrophotometrically using a DeNovix DS-11 FX spectrophotometer (DeNovix Inc., Wilmington, DE, United States).

### Multilocus sequence typing of *Campylobacter* isolates

Amplification of seven housekeeping genes was performed according to the procedures described by Dingle et al. (2001) using the primer sets given in the *Campylobacter* MLST website.^1^ All PCR products were purified with a DNA Clean & Concentrator™-5 kit (Zymo Research, Irvine, CA, United States). The purified PCR products were sent to the core facilities at the USDA ARS Genomics and Bioinformatics Research Unit (Stoneville, MS, United States) for DNA sequencing with an ABI 3730xl Genetic Analyzer (Thermo Fisher Scientific, Foster City, CA, United States) using a BigDye terminator v.3.1 Chemistry. Allelic profile, sequence type (ST) and clonal complex (CC) were assigned to the isolates using the allelic profile query function in the MLST database. Minimum spanning tree (MST) of MLST allelic differences was generated using BioNumerics (version 7.6; Applied Maths, Austin, TX, United States).

### Antimicrobial susceptibility test

Antimicrobial susceptibility of *C. jejuni* and *C. coli* isolates was determined using a Sensititre™ system (Thermo Fisher Scientific) according to the manufacturer’s instructions described previously (Yeh et al., 2022). Sensititire™ *Campylobacter* CAMPY AST plates were used in this study (Thermo Fisher Scientific). The results were read photometrically using Sensititre™ Vizion™ Digital MIC Viewing System (Thermo Fisher Scientific) in associated with the SWIN software (version 3.3). Quality control was performed using *C. jejuni*, ATCC 33560. The breakpoints for *Campylobacter* resistance were interpreted according to the guidelines from Clinical and Laboratory Standards Institute M45, 3rd Edition (Clinical and Laboratory Standards Institute (CLSI), 2015) as follows: azithromycin, ≥8 μg/mL; erythromycin, ≥32 μg/mL; gentamicin, ≥8 μg/mL; tetracycline, ≥16 μg/mL; ciprofloxacin, ≥4 μg/mL; florfenicol, ≥16 μg/mL; nalidixic acid, ≥32 μg/mL; and clindamycin, ≥8 μg/mL.

### Molecular detection of antibiotic resistance-associated genes

Resistance-associated genes of tetracycline, quinolones and macrolides in resistant isolates were determined. For tetracycline, the presence of the *tetO* gene was determined as described previously by Gibreel et al. (2004). Primers DMT 1 and DMT 2 (Table 1) were used to amplify a 559-bp product of the *tetO* gene in *Campylobacter* genomes. The mismatch amplification mutation assay (MAMA-PCR) was used to detect point mutations at Thr-86-Ile in QRDR of the *gyrA* gene (Zirnstein et al., 1999) and Ala-2074-Cys and Ala-2075-Gly in 23S rRNA gene (Alonso et al., 2005) for quinolone- and erythromycin-resistant isolates, respectively. Also, the *ermB* gene was used for screening the erythromycin resistant isolation according to the protocol described by Zhou et al. (2016). Primer sequences for PCR amplification are listed in Table 1.

**Table 1:** Oligonucleotide primers used in this study.

Virulence trait/function	Target gene	Sequence (5' – 3')	Annealing temperature	Product size (bp)	Reference
Motility	*flaA*	F: GGATTTCGTATTAACACAAATGGTGC R: CTGTAGTAATCTTAAACATTTTG	48 °C	1,700	Campynet
	*flhA*	F: GGAAGCGGCACTTGGTTTGC R: GCTGTGAGTGAGATTATAGCAGC	55 °C	735	Müller et al. (2006)
Adhesion and colonization	*cadF*	F: TGGAGGGTAATTTAGATATG R: CTAATACCTAAAGTTGAAAC	45 °C	400	Konkel et al. (1997)
	*docA*	F: ATAAGGTGCGGTTTTGGC R: GTCTTTGCAGTAGATATG	50 °C	725	Müller et al. (2006)
	*racR*	F: GATGATCCTGACTTTG R: TCTCCTATTTTTACCC	50 °C	584	Datta et al. (2003)
	*virB11*	F: GAACAGGAAGTGGAAAAACTAGC R: TTCCGCATTGGGCTATATG	56 °C	708	Bacon et al. (2000)
Cytotoxin production	*cdtA*	F: CCTTGTGATGCAAGCAATC R: ACACTCCATTTGCTTTCTG	55 °C	370	Hickey et al. (2000)
	*cdtB*	F: CAGAAAGCAAATGGAGTGTT R: AGCTAAAAGCGGTGGAGTAT	57 °C	620	Datta et al. (2003)
	*cdtC*	F: CGATGAGTTAAAACAAAAAGATA R: TTGGCATTATAGAAAATACAGTT	55 °C	182	Datta et al. (2003)
	*wlaN*	F: TGCTGGGTATACAAAGGTTGTG R: AATTTTGGATATGGGTGGGG	60 °C	330	Müller et al. (2006)
	*ceuE*	F: CCTGCTCGGTGAAAGTTTTG R: GATCTTTTTGTTTTGTGCTGC	57°C	794	Bang et al. (2003)
Invasiveness	*ciaB*	F: TTTCCAAATTTAGATGATGC R: GTTCTTTAAATTTTTCATAATGC	50 °C	1,165	Müller et al. (2006)
	*iam*	F: GCGCAAAATATTATCACCC R: TTCACGACTACTATGCGG	56 °C	518	Carvalho et al. (2001)
Erythromycin resistance	*ermB*	F: CAGGTAAAGGGCATTTAACGACG R: CATCTGTGGTATGGCGGGTAAG	58 °C	738	Zhou et al. (2016)
23S rRNA at position 2074 23S rRNA at position 2075	23SRNA-F ERY2074R ERY2075R	F: TTAGCTAATGTTGCCCGTACCG R: AGTAAAGGTCCACGGGGTCTGG R: TAGTAAAGGTCCACGGGGTCGC	59 °C	485 485	Alonso et al. (2005)
*tetO*	DMT 1	F:5GGCGTTTTGTTTATGTGCG 3 R:5ATGGACAACCCGACAGAAGC3		559	Gibreel et al. (2004)
MAMA-PCR (*gyrA* mutation) *C. jejuni*	CampyMAMAgyrA1 CampyMAMAgyrA5	F: TTTTTAGCAAAGATTCTGAT CAAAGCATCATAAACTGCAA		265	Zirnstein et al. (1999)
MAMA-PCR (*gyrA* mutation) *C. coli*	GZgyrACcoli3F CampyMAMAgyrA8	F:TATGAGCGATATTATCGGTC R:TAAGGCATCGTAAACAGCCA		192	Zirnstein et al. (1999)

### Detection of virulence-associated genes

*Campylobacter* isolates were screened for the presence of some virulence determinants by PCR, including the genes responsible for motility (*flaA* and *flhA*), adhesion and colonization (*cadF, docA, racR,* and *virB11*), cytotoxin production (*cdtA*, *cdtB, cdtC*, *ceuE,* and *wlaN*) and invasion-associated markers (*iam* and *ciaB*). Primer sequences and protocol for PCR amplification of the above virulence factors are listed in Table 1.

### Statistical analysis

To determine if the differences in the frequencies of isolate recovery was significant among the examined sources as well as frequencies of virulence genes among the examined isolates, these frequencies were used as inputs to create contingency tables and the significance was determined by Chi-square (*X*^2^) test, with a cutoff level for *p*-value equal to 0.05. The results of resistance phenotypes and frequencies of virulence genes were converted into binary data (0/1), where the presence of a virulence gene received scores of 1, whereas susceptibility to antimicrobials and the absence of a virulence gene received scores of 0. To determine the association of resistance phenotypes and virulence genes to sequence types (STs) among the examined *Campylobacter*, a heatmap with hierarchical clustering based on the binary data (0/1) of antimicrobial resistance and virulence genes was created using the package “pheatmap” in R software (version 217 3.4.2).

## Results

### Genetic diversity of *Campylobacter* isolates using MLST

**Figure 1:**
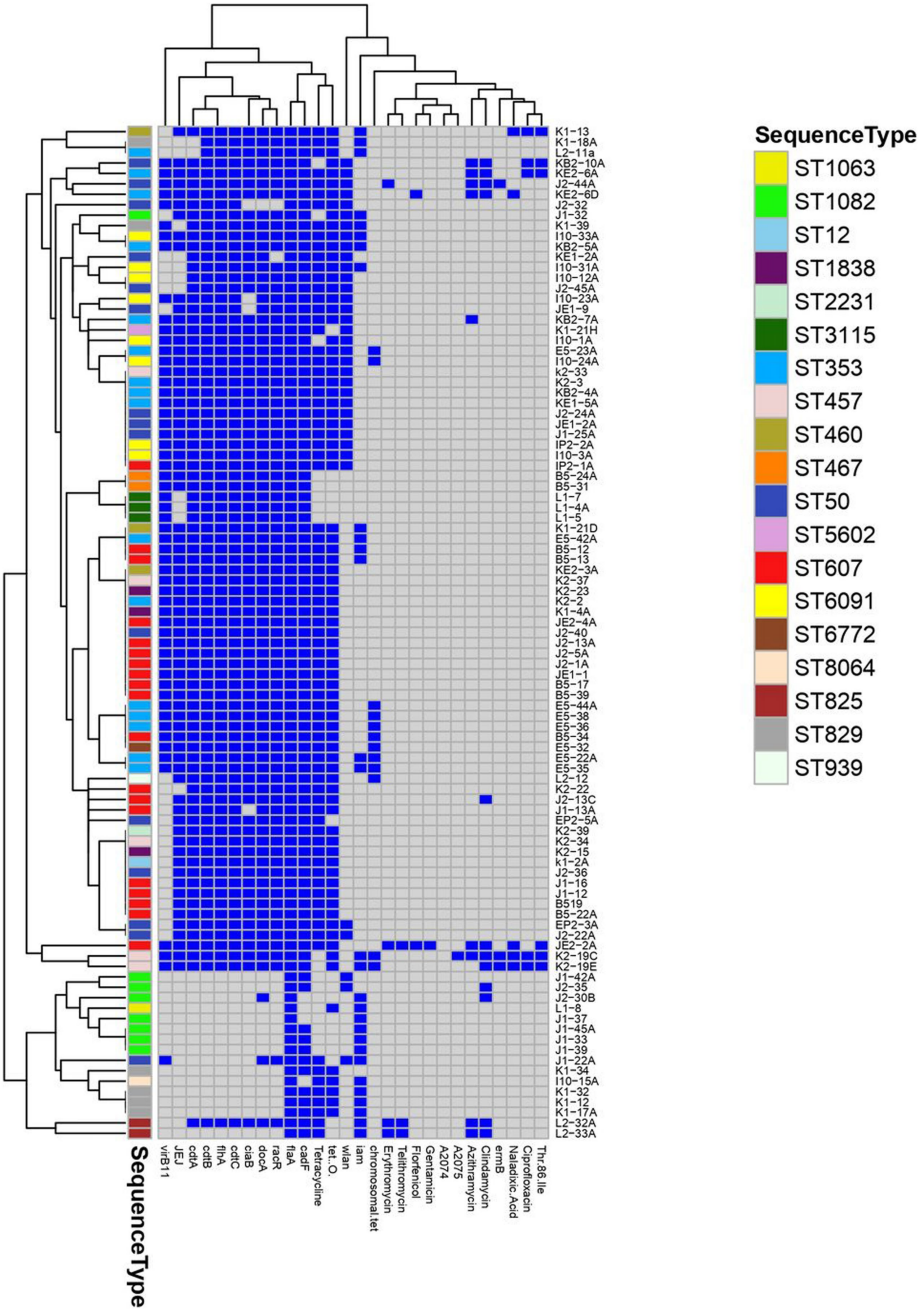
A heatmap supported by a dendrogram showing the distribution of antimicrobial resistance phenotypes, resistance and virulence genes among the examined *Campylobacter* assigned to various multilocus sequence types (ST). Dark blue squares indicate the presence of virulence and resistance genes and phenotypic resistance; gray squares indicate absent genes and phenotypic susceptibility.

MLST analysis showed high genetic diversity among both *C. jejuni* and *C. coli* isolates (Figure 1). A total of 19 different STs were identified: 14 for *C. jejuni* and five for *C. coli* (Table 2). The STs found in *C. jejuni* included ST-607 (*n* = 19), ST-353 (*n* = 16), ST-50 (*n* = 15), ST-6091 (*n* = 8), ST-457 (*n* = 5), ST-460 (*n* = 3), ST-1838 (*n* = 3), ST-3115 (*n* = 3), ST-467 (*n* = 2), ST-12 (*n* = 1), ST-939 (*n* = 1), ST-2231 (*n* = 1), ST-5602 (*n* = 1) and ST-6772 (*n* = 1). *C. jejuni* isolates from broiler feces showed the most diversity, including 11 STs, followed by seven STs found in broiler cecae. Further, 12 *C. jejuni* STs could be assigned to six previously described CCs (CC21, CC607, CC353, CC49, CC354, and CC460), whereas two (ST-5602 and ST-6091) belonged to undefined CCs. The STs found in *C. coli* were assigned to a single previously described CC828 included ST-8064 (*n* = 1), ST-829 (*n* = 6), ST-825 (*n* = 2), ST-1082 (*n* = 8) and ST-1063 (*n* = 1).

**Table 2:** Distribution of *C. jejuni* and *C. coli* MLST (ST) according to samples’ sources.

Source of isolates	*C. jejuni*														*C. coli*				
	CC-353							CC-354	CC-607	CC-21	CC-460	CC-49	UA	UA	CC-828				
	ST-353	ST-2231	ST-1838	ST-12	ST-457	ST-6772	ST-939	ST-3115	ST-607	ST-50	ST-460	ST-467	ST-5602	ST-6091	ST-8064	ST-825	ST-1082	ST-829	ST-1063
Broiler ceca (16)	1				2	1			1	1		1		2		2	3	2	
Broiler feces (37)	5		3	1			1	2	10	4	2	1	1	5	1			1	
Carcass rinse (10)	2	1			1				1	2							2	1	
Whole carcass rinse final (6)	2									2							2		
Broiler soil (10)^a^					2			1	3								1	2	1
Layer feces (7)	1								3	2	1								
Pig feces (4)									1	2				1					
Cow feces (2)	2																		
Cow soil (2)^a^	1									1									
Layer soil (3)^a^	2									1									
Total	16	1	3	1	5	1	1	3	19	15	3	2	1	8	1	2	8	6	1

^a^Soil sources indicate isolates recovered from the pasture topsoil in the same area the fecal samples were collected on that sampling day.

UA, undefined CCs.

Thermophilic isolates used in this study were originating from nine pastured poultry and livestock raised flocks, including broiler feces, broiler soil, broiler ceca, whole carcass rinse, pig feces, layer feces, layer soil, final whole carcass rinse, cow feces, and cow soil. By studying the frequency distribution of the recovered *C. jejuni* and *C. coli* from different sources, a significant (*p* < 0.05) associations of isolate recovery to the examined sources was obvious (Supplementary Table S1).

Concerning the distribution of *C. jejuni* and *C. coli* STs according to the source of samples, 7STs were detected belonging to CC-353 which was the most frequent clonal complex identified including ST-353 from broiler ceca, broiler feces, whole carcass rinse, layer feces, cow feces, cow soil and layer soil, ST-2231 from carcass rinse, ST-1838 from broiler feces (3), ST-12 from broiler feces (1), ST-457 was detected from carcass rinse and broiler soil, ST-6772 was detected from broiler ceca (1) and ST-939 was detected from broiler feces. Regarding CC 607, only ST-607 from broiler ceca, broiler feces, carcass rinse, broiler soil, layer feces and pig feces was identified. From CC-21 only ST- 50 was detected from broiler ceca, broiler feces, carcass rinse, whole carcass rinse, broiler soil, layer feces and pig feces. In addition to CC 460, ST- 460 was identified from broiler feces and layer feces and CC49 from which ST-467 was detected from broiler ceca and broiler feces. Furthermore, two STs not assigned to any clonal complex were also identified including ST-5602 from broiler feces and ST-6091 was detected from broiler ceca, broiler feces and pig feces. Regarding *C. coli* only CC-828 were detected and 5 STs were identified including ST- 8064 (broiler feces), ST- 825 (broiler ceca), ST-1082 (broiler ceca, carcass rinse, whole carcass rinse, broiler soil), ST-829 (broiler ceca, broiler feces, carcass rinse, broiler soil) and ST-1063 from broiler soil (Table 2).

### Antimicrobial susceptibility of *Campylobacter jejuni* and *Campylobacter coli* isolates

Frequency of antibiotic resistance of the *C. jejuni* and *C. coli* isolates to various antibiotics is presented in Table 3 and Figure 1. In total, 75 (95%) of the *C. jejuni* isolates were resistant to various numbers of antibiotics tested. Fifty-nine isolates (75%) were resistant to tetracycline alone, four isolates (5%) were resistant to two antibiotics (azithromycin and tetracycline), and another four (5%) were pan susceptible to all nine antibiotics tested. Twelve isolates were resistant to at least three antibiotics, and therefore considered multi-drug resistant (MDR), with one isolate showing resistance to eight antibiotics (azithromycin, clindamycin, erythromycin, florfenicol, gentamicin, telithromycin, tetracycline, and nalidixic acid). For *C. coli*, five (28%) and two (11%) isolates were resistant to tetracycline and clindamycin, respectively. However, two (11%) *C. coli* isolates were resistant to five antibiotics (tetracycline, azithromycin, clindamycin, erythromycin, and telithromycin). All *C. coli* isolates were sensitive to the quinolone class antibiotics (nalidixic acid and ciprofloxacin).

**Table 3:** Results of antimicrobial susceptibility testing for the examined *Campylobacter* isolates.

Antimicrobials	Class	*C. jejuni* (*n*=79)	*C. coli* (*n*=18)
		Resistant No. (%)	Resistant No. (%)
Tetracycline	Tetracycline	59 (74.68%)	5 (27.78%)
Clindamycin	Lincomycin	0	2 (11.11%)
Tetracycline, Azithromycin	Tetracycline, Macrolide	1 (1.27%)	0
Tetracycline, Clindamycin	Tetracycline, Lincomycin	4 (5.06%)	0
Tetracycline, Erythromycin	Tetracycline, Macrolide	1 (1.27%)	0
Tetracycline, Gentamicin	Tetracycline, Aminoglycoside	1 (1.27%)	0
Tetracycline, Ciprofloxacin, Nalidixic acid	Tetracycline, Quinolone	1 (1.27%)	0
Tetracycline, Clindamycin, Erythromycin	Tetracycline, Lincomycin, Macrolide	1 (1.27%)	0
Azithromycin, Ciprofloxacin, Clindamycin	Macrolide, Quinolone, Lincomycin	1 (1.27%)	0
Ciprofloxacin, Clindamycin, Nalidixic acid	Quinolone, Lincomycin	1 (1.27%)	0
Tetracycline, Azithromycin, Ciprofloxacin, Clindamycin	Tetracycline, Macrolide, Quinolone, Lincomycin	1 (1.27%)	0
Tetracycline, Azithromycin, Clindamycin, Erythromycin	Tetracycline, Macrolide Lincomycin	1 (1.27%)	0
Azithromycin, Ciprofloxacin, Clindamycin, Nalidixic acid	Macrolide, Quinolone, Lincomycin	1 (1.27%)	0
Tetracycline, Azithromycin, Clindamycin, Erythromycin, Telithromycin	Tetracycline, Lincomycin, Macrolide, Ketolide	0	2 (11.11%)
Tetracycline, Azithromycin, Clindamycin, Florfenicol, Nalidixic acid	Tetracycline, Macrolide, Lincomycin, Amphenicol, Quinolone	1 (1.27%)	0
Tetracycline, Azithromycin, Clindamycin, Erythromycin, Florfenicol, Gentamicin, Telithromycin, Nalidixic acid	Tetracycline, Macrolide, Lincomycin, Amphenicol, Aminoglycoside, Ketolide, Quinolone	1 (1.27%)	0
Total		75 (95%)	9 (50%)

### Detection of antimicrobial resistance mechanisms

The *tetO* gene that is responsible for tetracycline-resistant was detected in 80 isolates (82.5%) including 71 (89.9%) for *C. jejuni* and 9 (50%) for *C. coli*. Interestingly, the *tetO* gene was not detected in five phenotypically resistant isolates, but was detected from six phenotypically sensitive strains. The point mutation in *gyrA* responsible for quinolone resistance of *C. jejuni* (*n* = 5) and *C. coli* (*n* = 1) isolates was detected using MAMA-PCR. All phenotypically resistant isolates had a point mutation in the *gyrA*. For erythromycin-resistant isolates, *ermB* was detected in three isolates. The mutated A2075 was found in one isolate, while the A2074 mutation could not be identified.

### Distribution of virulence genes

Analysis of virulence gene distribution among *C*. *jejuni* and *C. coli* isolates revealed that all 97 *Campylobacter* isolates harbored the virulence genes tested (Table 4). All isolates contained the *flaA* gene (100%) and the other genes were detected in a high prevalence rate, including *flhA* (84.5%; 82/97), *cadF* (84.5%; 82/97), *docA* (85.6%, 83/97), *ciaB* (80.4%, 79/97), *racR* (83.5%, 81/97), *cdtC* (84.5%; 82/97), *cdtB* (84.5%, 82/97), *cdtA* (82.5%, 80/97), *ceuE* (72.2, 70/97), and *VirB11* (58.8%, 57/97). On the other hand, the *wlaN* gene was detected in only 35 isolates (36.1%) and *iam* gene was found in only 29 isolates (29.9%). In addition, the frequency of genes encoding adhesion and colonization factors in *C. jejuni* was significantly higher than that in *C. coli* (Table 4).

**Table 4:** Prevalence of virulence gene markers from *C. jejuni* and *C. coli* isolates.

Virulence factors	Target genes	*C. jeju*ni (*n*=79) (%)	*C. coli* (*n*=18) (%)	Total (*n*=97) (%)
Motility	*flaA*	79 (100%)	18 (100%)	97 (100%)
	*flhA*	78 (98.7%)	4 (22.2%)	82 (84.5%)
	*p* value	0.0054*		
Adhesion and colonization	*cadF*	79 (100%)	3 (16.7%)	82 (84.5%)
	*docA*	78 (98.7%)	5 (27.8%)	83 (85.6%)
	*racR*	77 (97.5%)	4 (22.2%)	81 (83.5%)
	*virB11*	56 (70.9%)	1 (5.6%)	57 (58.8%)
	*p* value	**0.645**		
Cytotoxin production	*cdtA*	77 (97.5%)	3 (16.7%)	80 (82.5%)
	*cdtB*	78 (98.7%)	4 (22.2%)	82 (84.5%)
	*cdtC*	78 (98.7%)	4 (22.2%)	82 (84.5%)
	*ceuE*	69 (87.3%)	1 (5.6%)	70 (72.2%)
	*wlaN*	31 (3.8%)	4 (22.2%)	35(36.1%)
	*p* value	**0.2396**		
Invasiveness	*ciaB*	74 (93.7%)	4 (22.2%)	78(80.4%)
	*iam*	14 (17.7%)	15 (83.3%)	29 (29.9%)
	*p* value	0.00001*		

*Indicates statistical significance. Bold values are just indicates the *p* values after statistical analysis.

## Discussion

Within the poultry industry, concerns have been expressed over the microbiological safety of pasture-raised poultry products despite consumer confidence in these types of production. The continuous exposure of the flocks to the pasture environment increases the possibility of contact with other sources of *Campylobacter* such as wild birds, insects, etc. (Berg, 2001). Due to the growing preference of this type of meat product, the question of whether the welfare benefits for this type of production is aligned with appropriate food safety should be explored. As a result, the current study was carried out to explore the genetic relatedness, virulence, and antimicrobial susceptibility of thermophilic *Campylobacter* by characterizing 97 isolates from pasture-raised poultry farms and the following processing operations.

*Campylobacter* sequence-based genotyping techniques yield data that is consistent across host sources, reproducible, and suitable for population genetic study (Dingle et al., 2001). Multi-locus sequence typing (MLST) identifies clonal complexes and links *Campylobacter* species to specific animal sources (Dingle et al., 2002; Colles et al., 2008). In this study, *Campylobacter* genotypes identified by MLST were diverse based on the number of samples taken from each flock. These results are in an agreement with the results reported by Colles et al. (2010) who found a great diversity in *Campylobacter* genotypes isolated from free-range broiler flocks. However, Bull et al. (2006) and Lindmark et al. (2006) reported a lower ST diversity of up to three STs within housed flocks. These discrepant findings highlight the importance of collecting large numbers of samples from a flock in order to identify the full range of variability within a flock. The most common clonal complexes CC607, CC21 and CC353 were predominant among *C. jejuni* strains in our study. These CCs were reported also as the most common CCs identified from human samples in various geographic regions (Dingle et al., 2001; de Haan et al., 2010; Smid et al., 2013). On the ST level, ST-353 and ST-50 were reported also as the most widely distributed STs among human and broiler *C. jejuni* isolates (Harvala et al., 2016; Elhadidy et al., 2018). These results highlight the importance of poultry sources for human campylobacteriosis.

The presence of thirteen virulence genes was investigated by PCR to confirm the pathogenic potential of these isolates. Significant differences in the occurrence of virulence genes were observed, *C. jejuni* isolates had a higher virulence potential than *C. coli* isolates. These results are in an agreement with those reported by Casabonne et al. (2016) and Wieczorek et al. (2013) from conventionally raised broiler flocks. Our results showed that the *flaA* gene was detected in all strains, and the *flhA* gene was found in most of the isolates examined. Similar findings are also reported by Rossler et al. (2020) that *flaA* and *flhA* genes were detected in all their isolates collection. Mobility of *Campylobacter*, involving the coordination of many genes (such as *flaA* and *flhA*), is important for passage through the stomach and gut (Gilbreath et al., 2011). The presence of *flaA* and *flhA* genes in a high proportion of the isolates examined suggests that motility and virulence mechanism are synchronized during *Campylobacter* pathogenesis (Wieczorek et al., 2015; Zhang et al., 2016; Frazão et al., 2017; Rossler et al., 2020).

The *Campylobacter* adhesion to fibronectin F (*cadF*) gene, encoding an adhesin and fibronectin-binding protein that involves in the process of invasion and influences the microfilament organization in host cells (Zhang et al., 2016), was also detected in most of our isolates (Table 4). Similar observations have been reported that the high frequency of the *cadF* gene in *Campylobacter* species was detected from poultry productions and processing operations (Rozynek et al., 2005; Frazão et al., 2017; Rossler et al., 2020). Additionally, Ziprin et al. (1999) demonstrated that *Campylobacter* *cadF*-negative strains are not able to colonize in chicken gastrointestinal tract. Therefore, the *cadF* gene product may play a similar function in human pathogenesis and causing disease.

The Guillain-Barré syndrome associated gene (*wlaN*) and its gene product have ganglioside-like structures and is responsible for specific lipooligosaccharides (LOS) synthesis (Hermans et al., 2011). This LOS synthesis is thought to be involved in the development of Guillain-Barré and Miller-Fischer syndromes after *C. jejuni* infection (Gilbert et al., 2000; Linton et al., 2000). The presence of this gene in *Campylobacter* may increase the risk for suffering post-neurological conditions. Our findings indicate its presence in 36.1% of the total thermophilic *Campylobacter* isolates, which is in line with many previous studies in conventional poultry management systems (Talukder et al., 2008; Koolman et al., 2015; Wieczorek et al., 2018).

*Campylobacter* toxins are important virulence marker determinants. One of the toxin groups is the cytolethal distending cytotoxins, which are encoded by the *cdt* genes and form polycistronic *cdt* operons. The gene products include CdtA, CdtB, and CdtC cytotoxins, which are toxic to host enterocytes (Carvalho et al., 2013). These cytotoxins play important roles in development of diarrhea by interfering with the proliferation and differentiation of intestinal crypt cells (Scuron et al., 2016). The three subunits are required for the full activity of the toxins (Lapierre et al., 2016). CdtB displays enzymatic Dnase activity resulting in cell-cycle arrest and cell death, while CdtA and CdtC are responsible for the translocation of CdtB across the target cell membrane (Lara-Tejero and Galan, 2001). In this study, these toxin genes were detected in majority of *C. jejuni* isolates (98.7–97.5%).

*Campylobacter* survival in the digestive tract is highly dependent on the *ciaB* gene. This gene can secret a CiaB protein that is responsible for the invasion and colonization of this microorganism in chicken intestines (Hermans et al., 2011). Among *Campylobacter* isolates, it was found in a frequency of 80.4%. Similarly, in conventionally reared broilers, the *ciaB* gene was detected in a similar prevalence by Raeisi et al. (2017) and Wieczorek et al. (2018). Because the *ciaB* gene is important in the early stages of colonization, their removal may causes bacterial failure to survive the stress of passage through the gut followed by colonization failure (Ziprin et al., 2001). Additionally, regulatory protein R (*racR*) gene and its gene product regulate temperature during growth and colonization of this microorganism in the hosts. The prevalence of *racR* in our study is 83.5%, which is similar to that reported in conventional broiler flocks by Datta et al. (2003) and Talukder et al. (2008); however, Hanning et al. (2012) demonstrated a lower *racR* prevalence rate (34%) in a pasture-raised broiler flock study.

The enterochelin binding lipoprotein encoded by siderophore transport (*ceuE*), which has an important role in virulence and regulation of the siderophore transport system (Hermans et al., 2011), was detected in 72.2% of the *Campylobacter* isolates.

The use of antibiotics, either overuse or abuse, in food animals contributes to the establishment of antimicrobial resistance (AMR) in commensal and zoonotic enteric bacteria (Varga et al., 2009; van Boeckel et al., 2015). To prevent the spread of AMR *Campylobacter* through the food chain, it is critical to continuously monitor its antimicrobial resistance and resistance mechanisms. In this study, five (5.2%) *Campylobacter* isolates were resistant to quinolones and fluroquinolones [nalidixic acid (*n* = 4) and ciprofloxacin (*n* = 1)]. The lower resistance of quinolones and fluoroquinolone-resistant isolates in this study may be related to that the U.S. Food and Drug Administration (FDA) banned the use of fluoroquinolones in poultry production in the United States in 2005 (Griggs et al., 2005). However, other studies argued that the FDA’s restriction on fluoroquinolones in chicken production may not be enough to mitigate the resistant *Campylobacter* in poultry products, because fluoroquinolone-resistant *Campylobacter* was found in persistent pollutants of poultry products even after discontinuous on-farm fluoroquinolone use (Price et al., 2007). Monitoring the prevalence of resistant strains in chicken flocks, production facilities, consumer poultry products, and human diseases is therefore crucial in order to accurately evaluate the effectiveness of this policy. The low frequency of resistance to quinolones and fluoroquinolones in this study may also related to the fact that antibiotics were not utilized by any farms during the duration of this study. Luangtongkum et al. (2006) reported a significant difference between antimicrobial resistance rates of <2% vs. 46–67% in organic and conventional raised poultry farms, respectively.

The *Campylobacter* isolates that displayed resistant to quinolones and fluroquinolones [ciprofloxacin (MIC = 8 μg/mL) and nalidixic acid (MIC = 64 μg/mL), respectively] were further examined for the presence of the most common mutation site. A point mutation at position 86 leading to threonine replacement by isoleucine was detected in the QRDR of the *gyrA* gene from our isolates. A MAMA-PCR was used to determine the presence of this type of mutation (Zirnstein et al., 1999; Payot et al., 2004). In this protocol, a conserved forward primer, CampyMAMA*gyrA*1, and a reverse mutation detection primer, CampyMAMA*gyrA*5 were used to generate a 265-bp PCR product, indicating the presence of the Thr-86-Ile (ACA to ATA) mutation in the *C. jejuni gyrA* gene. This method was used as an alternative to nucleotide sequencing because it is not accessible in ordinary microbiology laboratories. Our results revealed that this mutation was found among all phenotypically resistant isolates. In contrast, this mutation was found to be absent in some quinolone resistance isolates, leading researchers to speculate that it could be linked to alternative resistance mechanisms (Bolton et al., 2013; Elhadidy et al., 2018; Yeh et al., 2022).

Emergence of resistance to erythromycin by *Campylobacter* isolates has been reported (Deng et al., 2015; Liu et al., 2019; Jehanne et al., 2021). Resistance of *Campylobacter* to this macrolide is chromosomally mediated, most commonly due to a shift in the target site on the 23S rRNA subunit. These mutations have been identified at locations of 2074 and 2075 (Vacher et al., 2003). The transitory mutation A2075G is the most prevalent among erythromycin-resistant *Campylobacter* isolates, while the A2074C mutation is less identified among the resistant strains (Vacher et al., 2003). In erythromycin resistant isolates in this study (MIC >64 μg/mL), A2075G was detected in one isolate, while A2074G could not be identified from any isolate. Additionally, *ermB* was found in three out of nine erythromycin-resistant isolates, while Elhadidy et al. (2020) could not identify *ermB* gene from any erythromycin-resistant *Campylobacter* isolates. These findings on the molecular basis of macrolide resistance in *Campylobacter* revealed the importance of additional resistance mechanisms in *Campylobacter* encoding erythromycin resistance such as CmeABC is a multi-drug efflux pump system broadly distributed in *Campylobacter*, representing an important mechanism for antibiotic resistance (Lin et al., 2002).

Interestingly, one isolate in this study was gentamicin resistant (MIC>32 μg/mL). This result is congruent with that of Luangtongkum et al. (2006), who reported that none of the *Campylobacter* species isolated from conventionally or organically raised broilers were gentamicin resistant. Similarly, Giacomelli et al. (2014) and Elhadidy et al. (2018) could not identify gentamicin resistant isolates among *Campylobacter* isolates from poultry in Italy and Belgium, respectively. On the other hand, Saenz et al. (2000) found a 25% prevalent rate of gentamicin resistant isolates from broilers in Spain. The common low prevalence of gentamicin resistance may contribute to few usages of this antibiotic during the poultry production (Saenz et al., 2000; Roth et al., 2019).

Our results showed the high prevalence of tetracycline resistance among the isolates (MIC >64 μg/mL) (Table 3). These findings are also reported by other researchers from Kenya, Finland, Iraq, Poland, and USA (Luangtongkum et al., 2006; Nguyen et al., 2016; Pohjola et al., 2016; Wieczorek et al., 2018; Shakir et al., 2021) where *C. jejuni* and *C. coli* were isolated from small scale and backyard chicken flocks. In addition, Bailey et al. (2019) found that tetracycline resistances in organic farms were more common than the conventional farms. The reports have demonstrated that the plasmid-encoded *tet (O)* gene is responsible for tetracycline resistance in *Campylobacter* (Gibreel et al., 2004; Wozniak-Biel et al., 2018; Elhadidy et al., 2019), and this gene can be horizontally transferred between *C. jejuni* and *C. coli* isolates in the intestines of food animals and humans (Kim et al., 2010). Interestingly, the presence of phenotypic tetracycline resistant isolates that did not harbored *tet (O)* gene may be related to the genetic inactivated of efflux pumps (Jeon et al., 2011). The high rates of resistance reported for tetracycline could be attributed to the overuse during the poultry production (Giacomelli et al., 2014).

In conclusion, MLST analysis showed high genetic diversity among both *C. jejuni* and *C. coli* isolates. The identified STs were reported also as the most common STs identified from human samples in various geographic regions. These results highlight the importance of poultry sources for human campylobacteriosis. Additionally, *Campylobacter* isolated from pasture-raised poultry flocks from this study were generally consistent with *Campylobacter* previously isolated from conventionally reared broiler flocks in regard to ST prevalence and diversity, antibiotic resistance patters, and virulence. Thus, in terms of public health risk of campylobacteriosis, these results indicate that pasture-raised poultry products appear to be equivalent conventionally reared products, but still represents a potential zoonotic source of *Campylobacter* that requires further investigation.

## Data availability statement

The datasets presented in this study can be found in online repositories. The names of the repository/repositories and accession number(s) can be found in the article/Supplementary material.

## Author contributions

AA: Conceptualization, Data curation, Formal analysis, Investigation, Methodology, Visualization, Writing – original draft, Writing – review & editing. H-YY: Conceptualization, Data curation, Formal analysis, Funding acquisition, Investigation, Project administration, Supervision, Writing – original draft, Writing – review & editing. HR: Data curation, Investigation, Methodology, Writing – review & editing. MR: Resources, Writing – review & editing.

## Footnotes

^1^https://pubmlst.org/bigsdb?db=pubmlst_campylobacter_seqdef

## References

[ref1] Aarestrup F. M. McDermott P. F. Wegener H. C. (2008). “Transmission of antibiotic resistance from food animals to humans” in Campylobacter. eds. Nachamkin I. Szymanski C. M. Blaser M. J. (Washington, D. C.: ASM Press), 645–665.

[ref2] Alonso R. Mateo E. Churruca E. Martinez I. Girbau C. Fernández-Astorga A. (2005). MAMA-PCR assay for the detection of point mutations associated with high-level erythromycin resistance in *Campylobacter jejuni* and *Campylobacter coli* strains. J. Microbiol. Methods 63, 99–103. doi: 10.1016/j.mimet.2005.03.013, PMID: 15927294

[ref9001] Bacon D. J. Alm R. A. Burr D. H. Hu L. Kopecko D. J. Ewing C. P.. (2000). Involvement of a plasmid in virulence of *Campylobacter jejuni 81-176*. Infect. Immun. 68, 4384–4390. doi: 10.1128/iai.68.8.4384-4390.2000 10899834 PMC98329

[ref3] Bailey M. A. Taylor R. M. Brar J. S. Corkran S. C. Velásquez C. Novoa Rama E.. (2019). Prevalence and antimicrobial resistance of *Campylobacter* from antibiotic-free broilers during organic and conventional processing. Poult. Sci. 98, 1447–1454. doi: 10.3382/ps/pey486, PMID: 30325456

[ref9002] Bang D. D. Moller Nielsen E. Scheutz F. Pedersen K. Handberg K. Madsen M. (2003). PCR detection of seven virulence and toxin genes of *Campylobacter jejuni* and *Campylobacter coli* isolates from Danish pigs and cattle and cytolethal distending toxin production of the isolates. J. Appl. Microbiol. 94, 1003–1014. doi: 10.1046/j.1365-2672.2003.01926.x 12752808

[ref4] Berg C. (2001). Health and welfare in organic poultry production. Acta Vet. Scand. 43, 1–9. doi: 10.1186/1751-0147-43-S1-S37 11995389

[ref5] Bolton D. J. (2015). *Campylobacter* virulence and survival factors. Food Microbiol. 48, 99–108. doi: 10.1016/j.fm.2014.11.017 25790997

[ref6] Bolton D. Patriarchi A. Fox Á. Fanning S. (2013). A study of the molecular basis of quinolone and macrolide resistance in a selection of *Campylobacter* isolates from intensive poultry flocks. Food Control 30, 222–226. doi: 10.1016/j.foodcont.2012.06.044

[ref7] Bull S. A. Allen V. M. Domingue G. Jørgensen F. Frost J. A. Ure R.. (2006). Sources of *Campylobacter* spp. colonizing housed broiler flocks during rearing. Appl. Environ. Microbiol. 72, 645–652. doi: 10.1128/AEM.72.1.645-652.2006, PMID: 16391102 PMC1352183

[ref9003] Caner V. Cokal Y. Cetin C. Sen A. Karagenc N. (2008). The detection of *hipO* gene by real-time PCR in thermophilic *Campylobacter* spp. with very weak and negative reaction of hippurate hydrolysis. Antonie Van Leeuwenhoek. 94, 527–532. doi: 10.1007/s10482-008-9269-4 18665452

[ref8] Carvalho A. F. da Silva D. M. Azevedo S. S. Piatti R. M. Genovez M. E. Scarcelli E. (2013). Detection of CDT toxin genes in *Campylobacter* spp. strains isolated from broiler carcasses and vegetables in São Paulo, Brazil. Braz. J. Microbiol. 44, 693–699. doi: 10.1590/s1517-83822013000300005, PMID: 24516435 PMC3910176

[ref9004] Carvalho A. C. T. Ruiz-Palacios G. M. Ramos-Cervantes P. Cervantes L.-E. Jiang X. Oickering L. K. (2001). Molecular characterization of invasive and noninvasive *Campylobacter jejuni* and *Campylobacter coli* isolates. J. Clin. Microbiol. 39, 1353–1359. doi: 10.1128/jcm.39.4.1353-1359.2001 11283056 PMC87939

[ref9] Casabonne C. Gonzalez A. Aquili V. Subils T. Balague C. (2016). Prevalence of seven virulence genes of *Campylobacter jejuni* isolated from patients with diarrhea in Rosario, Argentina. Int. J. Inf. Secur. 3:e37727. doi: 10.17795/iji-37727 26902216

[ref10] Centers for Disease Control and Prevention. (2022). Campylobacter. Available at: https://www.cdc.gov/campylobacter/faq.html (Accessed October 1, 2023).

[ref11] Clinical and Laboratory Standards Institute (CLSI) (2015) in M45: methods for antimicrobial dilution and disk susceptibility testing of infrequently isolated or fastidious bacteria. eds. Hindler J. A. Richter S. S.. 3rd ed 10.1086/510431 17173232

[ref12] Colles F. M. Jones T. A. McCarthy N. D. Sheppard S. K. Cody A. J. Dingle K. E.. (2008). *Campylobacter* infection of broiler chickens in a free-range environment. Environ. Microbiol. 10, 2042–2050. doi: 10.1111/j.1462-2920.2008.01623.x, PMID: 18412548 PMC2702501

[ref13] Colles F. M. McCarthy N. D. Sheppard S. K. Layton R. Maiden M. C. J. (2010). Comparison of *Campylobacter* populations isolated from a free-range broiler flock before and after slaughter. Int. J. Food Microbiol. 137, 259–264. doi: 10.1016/j.ijfoodmicro.2009.12.021 20071049 PMC3980632

[ref14] Conner D. S. Campbell-Arvai V. Hamm M. W. (2008). Consumer preferences for pasture-raised animal products: results from Michigan. J. Food Distrib. Res. 39, 12–25. doi: 10.22004/ag.econ.55972

[ref15] Datta S. Niwa H. Itoh K. (2003). Prevalence of 11 pathogenic genes of *Campylobacter jejuni* by PCR in strains isolated from humans, poultry meat and broiler and bovine faeces. J. Med. Microbiol. 52, 345–348. doi: 10.1099/jmm.0.05056-0, PMID: 12676874

[ref16] de Haan C. P. Kivisto R. Hakkinen M. Rautelin H. Hanninen M. L. (2010). Decreasing trend of overlapping multilocus sequence types between human and chicken *Campylobacter jejuni* isolates over a decade in Finland. Appl. Environ. Microbiol. 76, 5228–5236. doi: 10.1128/AEM.00581-10, PMID: 20543048 PMC2916457

[ref17] Deng F. Shen J. Zhang M. Wu C. Zhang Q. Wang Y. (2015). Constitutive and inducible expression of the rRNA methylase gene *erm* (B) in *Campylobacter*. Antimicrob. Agents Chemother. 59, 6661–6664. doi: 10.1128/AAC.01103-15, PMID: 26259800 PMC4576093

[ref9005] Dingle K. E. Colles F. M. Ure R. Wagenaar J. A. Duim B. Bolton F. J.. (2002). Molecular characterization of *Campylobacter jejune* clones: a basis for epidemiologic investigation. Emerg. Infect. Dis. 8, 949–955. doi: 10.3201/eid0809.02-0122 12194772 PMC2732546

[ref18] Dingle K. E. Van Den Braak N. Colles F. M. Price L. J. Woodward D. L. Rodgers F. G.. (2001). Sequence typing confirms that *Campylobacter jejuni* strains associated with Guillain-Barre and Miller-fisher syndromes are of diverse genetic lineage, serotype, and flagella type. J. Clin. Microbiol. 39, 3346–3349. doi: 10.1128/JCM.39.3.3346-3349.2001, PMID: 11526174 PMC88342

[ref19] Elhadidy M. Ali M. M. El-Shibiny A. Miller W. G. Elkhatib W. F. Botteldoorn N.. (2020). Antimicrobial resistance patterns and molecular resistance markers of *Campylobacter jejuni* isolates from human diarrheal cases. PLoS One 15:e0227833. doi: 10.1371/journal.pone.0227833 31951631 PMC6968864

[ref20] Elhadidy M. Miller W. G. Arguello H. Álvarez-Ordóñez A. Dierick K. Botteldoorn N. (2019). Molecular epidemiology and antimicrobial resistance mechanisms of *Campylobacter coli* from diarrhoeal patients and broiler carcasses in Belgium. Transbound. Emerg. Dis. 66, 463–475. doi: 10.1111/tbed.13046 30346650

[ref21] Elhadidy M. Miller W. G. Arguello H. Álvarez-Ordóñez A. Duarte A. Dierick K.. (2018). Genetic basis and clonal population structure of antibiotic resistance in *Campylobacter jejuni* isolated from broiler carcasses in Belgium. Front. Microbiol. 9:1014. doi: 10.3389/fmicb.2018.01014, PMID: 29867900 PMC5966580

[ref22] European Food Safety Authority (2021). The European Union one health 2019 zoonoses report. EFSA J. 19:e06406. doi: 10.2903/j.efsa.2021.6406, PMID: 33680134 PMC7913300

[ref23] Frazão M. R. Medeiros M. Duque S. Falcão J. P. (2017). Pathogenic potential and genotypic diversity of *Campylobacter jejuni*: a neglected food-borne pathogen in Brazil. J. Med. Microbiol. 66, 350–359. doi: 10.1099/jmm.0.000424, PMID: 28317494

[ref24] Ghareeb K. Awad W. A. Mohnl M. Schatzmayr G. Boehm J. (2013). Control strategies for *Campylobacter* infection in poultry production. Worlds Poult. Sci. J. 69, 57–76. doi: 10.1017/S0043933913000068

[ref25] Giacomelli M. Salata C. Martini M. Montesissa C. Piccirillo A. (2014). Antimicrobial resistance of *Campylobacter jejuni* and *Campylobacter coli* from poultry in Italy. Microb. Drug Resist. 20, 181–188. doi: 10.1089/mdr.2013.0110, PMID: 24320689

[ref26] Gibreel A. Tracz D. M. Nonaka L. Ngo T. M. Connell S. R. Taylor D. E. (2004). Incidence of antibiotic resistance in *Campylobacter jejuni* isolated in Alberta, Canada, from 1999 to 2002, with special reference to *tet* (O)-mediated tetracycline resistance. Antimicrob. Agents Chemother. 48, 3442–3450. doi: 10.1128/AAC.48.9.3442-3450.2004, PMID: 15328109 PMC514748

[ref27] Gilbert M. Brisson J.-R. Karwaski M.-F. Michniewicz J. Cunningham A.-M. Wu Y.. (2000). Biosynthesis of ganglioside mimics in *Campylobacter jejuni* OH 4384: identification of the glycosyltransferase genes, enzymatic synthesis of model compounds, and characterization of nanomole amounts by 600-MHz ^1^H and ^13^C NMR analysis. J. Biol. Chem. 275, 3896–3906. doi: 10.1074/jbc.275.6.3896, PMID: 10660542

[ref28] Gilbreath J. J. Cody W. L. Merrell D. S. Hendrixson D. R. (2011). Change is good: variations in common biological mechanisms in the epsilonproteobacterial genera *Campylobacter* and *Helicobacter*. Microbiol. Mol. Biol. Rev. 75, 84–132. doi: 10.1128/MMBR.00035-10, PMID: 21372321 PMC3063351

[ref29] Griggs D. J. Johnson M. M. Frost J. A. Humphrey T. Jorgensen F. Piddock L. J. V. (2005). Incidence and mechanism of ciprofloxacin resistance in *Campylobacter* spp. isolated from commercial chicken flocks in the United Kingdom before, during and after fluoroquinolone treatment. Antimicrob. Agents Chemother. 49, 699–707. doi: 10.1128/AAC.49.2.699-707.2005, PMID: 15673754 PMC547197

[ref30] Hanning I. Biswas D. Herrera P. Roesler M. Ricke S. C. (2012). Prevalence and characterization of *Campylobacter jejuni* isolated from pasture flock poultry. J. Food Sci. 75, M496–M502. doi: 10.1111/j.1750-3841.2010.01747.x, PMID: 21535562

[ref31] Harvala H. Rosendal T. Lahti E. Engvall E. O. Brytting M. Wallensten A.. (2016). Epidemiology of *Campylobacter jejuni* infections in Sweden, November 2011–October 2012: is the severity of infection associated with *C. jejuni* sequence type? Infect. Ecol. Epidemiol. 6:31079. doi: 10.3402/iee.v6.31079, PMID: 27059819 PMC4826459

[ref32] Hermans D. Van Deun K. Martel A. Immerseel F. Messens W. Heyndrickx M.. (2011). Colonization factors of *Campylobacter jejuni* in the chicken gut. Vet. Res. 42:82. doi: 10.1186/1297-9716-42-82, PMID: 21714866 PMC3156733

[ref9006] Hickey T. E. McVeigh A. L. Scott D. A. Michieluth R. E. Bixby A. Carroll S. A.. (2000). *Campylobacter jejuni* cytolethal distending toxin mediates release of interleukin-8 from intestinal epithelial cells. Infect. Immun. 68, 6535–6541. doi: 10.1128/iai.68.12.6535-6541.2000 11083762 PMC97747

[ref33] Hiett K. L. Stintzi A. Andacht T. M. Kuntz R. L. Seal B. S. (2008). Genomic differences between *Campylobacter jejuni* isolates identify surface membrane and flagellar function gene products potentially important for colonizing the chicken intestine. Funct. Integr. Genomics 8, 407–420. doi: 10.1007/s10142-008-0087-6 18592283

[ref34] Jehanne Q. Bénéjat L. Ducournau A. Domingues-Martins C. Cousinou T. Bessède E.. (2021). Emergence of erythromycin resistance methyltransferases in *Campylobacter coli* strains in France. Antimicrob. Agents Chemother. 65:e0112421. doi: 10.1128/AAC.01124-21, PMID: 34370579 PMC8522779

[ref35] Jeon B. Wang Y. Hao H. Barton Y. W. Zhang Q. (2011). Contribution of *Cme G* to antibiotic and oxidative stress resistance in *Campylobacter jejuni*. J. Antimicrob. Chemother. 66, 79–85. doi: 10.1093/jac/dkq418, PMID: 21081547 PMC3001851

[ref36] Kaakoush N. O. Castaño-Rodríguez N. Mitchell H. M. Man S. M. (2015). Global epidemiology of *Campylobacter* infection. Clin. Microbiol. Rev. 28, 687–720. doi: 10.1128/CMR.00006-15, PMID: 26062576 PMC4462680

[ref37] Kim J. M. Hong J. Bae W. Koo H. C. Kim S. H. Park Y. H. (2010). Prevalence, antibiograms, and transferable *tet (O)* plasmid of *Campylobacter jejuni* and *Campylobacter coli* isolated from raw chicken, pork, and human clinical cases in Korea. J. Food Prot. 73, 1430–1437. doi: 10.4315/0362-028X-73.8.1430, PMID: 20819352

[ref9007] Konkel M. E. Garvis S. G. Tipton S. L. Anderson Jr., D. E. Cieplak Jr., W. (1997). Identification and molecular cloning of a gene encoding a fibronectin-binding protein (CadF) from *Campylobacter jejuni*. Mol. Microbiol. 24, 953–963. doi: 10.1046/j.1365-2958.1997.4031771.x 9220003

[ref38] Koolman L. Whyte P. Burgess C. Bolton D. (2015). Distribution of virulence-associated genes in a selection of *Campylobacter* isolates. Foodborne Pathog. Dis. 12, 424–432. doi: 10.1089/fpd.2014.1883, PMID: 25826607

[ref39] Lapierre L. Gatica M. A. Riquelme V. Vergara C. Yañez J. M. San Martin B.. (2016). Characterization of antimicrobial susceptibility and its association with virulence genes related to adherence, invasion, and cytotoxicity in *Campylobacter jejuni* and *Campylobacter coli* isolates from animals, meat, and humans. Microb. Drug Resist. 22, 432–444. doi: 10.1089/mdr.2015.0055, PMID: 26779841

[ref40] Lara-Tejero M. Galan J. E. (2001). CdtA, CdtB, and CdtC form a tripartite complex that is required for cytolethal distending toxin activity. Infect. Immun. 69, 4358–4365. doi: 10.1128/IAI.69.7.4358-4365.2001, PMID: 11401974 PMC98507

[ref41] Lin J. Michel L. O. Zhang Q. (2002). CmeABC functions as a multidrug efflux system in *Campylobacter jejuni*. Antimicrob. Agents Chemother. 46, 2124–2131. doi: 10.1128/aac.46.7.2124-2131.2002, PMID: 12069964 PMC127319

[ref42] Lindmark H. Diedrich I. C. Andersson L. Lindqvist R. Engvall E. O. (2006). Distribution of *Campylobacter* genotypes on broilers during slaughter. J. Food Prot. 69, 2902–2907. doi: 10.4315/0362-028x-69.12.2902, PMID: 17186657

[ref43] Linton D. Gilbert M. Hitchen P. G. Dell A. Morris H. R. Wakarchuk A. A.. (2000). Phase variation of a beta-1,3 galactosyltransferase involved in generation of the ganglioside GM1-like lipo-oligosaccharide of *Campylobacter jejuni*. Mol. Microbiol. 37, 501–514. doi: 10.1046/j.1365-2958.2000.02020.x, PMID: 10931344

[ref9008] Linton D. Lawson A. J. Owen R. J. Stanley J. (1997). PCR detection, Identification to species level, and fingerprinting of *Campylobacter jejuni* and *Campylobacter coli* direct from diarrheic samples. J. Clin. Microbiol. 35, 2568–2572. doi: 10.1128/jcm.35.10.2568-2572.1997 9316909 PMC230012

[ref44] Liu D. Liu W. Lv Z. Xia J. Li X. Hao Y.. (2019). Emerging *erm* (B)-mediated macrolide resistance associated with novel multidrug resistance genomic islands in *Campylobacter*. Antimicrob. Agents Chemother. 63, e00153–e00119. doi: 10.1128/AAC.00153-19, PMID: 31085517 PMC6591595

[ref45] Luangtongkum T. Morishita T. Y. Ison A. J. Huang S. McDermott P. F. Zhang Q. (2006). Effect of conventional and organic production practices on the prevalence and antimicrobial resistance of *Campylobacter* spp. in poultry. Appl. Environ. Microbiol. 72, 3600–3607. doi: 10.1128/AEM.72.5.3600-3607.2006, PMID: 16672508 PMC1472326

[ref46] Ma L. Wang Y. Shen J. Zhang Q. Wu C. (2014). Tracking *Campylobacter* contamination along a broiler chicken production chain from the farm level to retail in China. Int. J. Food Microbiol. 181, 77–84. doi: 10.1016/j.ijfoodmicro.2014.04.023 24831929

[ref9009] Müller J. Schulze F. Muller W. Hanel I. (2006). PCR detection of virulence-associated genes in *Campylobacter jejuni* strains with differential ability to invade Caco-2 cells and to colonize the chick gut. Vet. Microbiol. 113, 123–129. doi: 10.1016/j.vetmic.2005.10.029 16300911

[ref47] Nguyen T. N. Hotzel H. Njeru J. Mwituria J. El-Adawy H. Tomaso H.. (2016). Antimicrobial resistance of *Campylobacter* isolates from small scale and backyard chicken in Kenya. Gut Pathog. 8:39. doi: 10.1186/s13099-016-0121-5, PMID: 27570543 PMC5002103

[ref48] Payot S. Avrain L. Magras C. Praud K. Cloeckaert A. Chaslus-Dancla E. (2004). Relative contribution of target gene mutation and efflux to fluoroquinolone and erythromycin resistance, in French poultry and pig isolates of *Campylobacter coli*. Int. J. Antimicrob. Agents 23, 468–472. doi: 10.1016/j.ijantimicag.2003.12.008 15120725

[ref49] Pohjola L. Nykäsenoja S. Kivistö R. Soveri T. Huovilainen A. Hänninen M. L.. (2016). Zoonotic public health hazards in backyard chickens. Zoonoses Public Health 63, 420–430. doi: 10.1111/zph.12247 26752227

[ref50] Price L. B. Lackey L. G. Vailes R. Silbergeld E. (2007). The persistence of fluoroquinolone-resistant *Campylobacter* in poultry production. Environ. Health Perspect. 115, 1035–1039. doi: 10.1289/ehp.10050, PMID: 17637919 PMC1913601

[ref51] Raeisi M. Khoshbakht R. Ghaemi E. A. Bayani M. Hashemi M. Seyedghasemi N. S.. (2017). Antimicrobial resistance and virulence-associated genes of *Campylobacter* spp. isolated from raw milk, fish, poultry, and red meat. Microb. Drug Resist. 23, 925–933. doi: 10.1089/mdr.2016.0183, PMID: 28177853

[ref52] Rossler E. Olivero C. Soto L. P. Frizzo L. S. Zimmermann J. Rosmini M. R.. (2020). Prevalence, genotypic diversity and detection of virulence genes in thermotolerant *Campylobacter* at different stages of the poultry meat supply chain. Int. J. Food Microbiol. 326:108641. doi: 10.1016/j.ijfoodmicro.2020.108641 32371295

[ref53] Roth N. Käsbohrer A. Mayrhofer S. Zitz U. Hofacre C. Domig K. J. (2019). The application of antibiotics in broiler production and the resulting antibiotic resistance in *Escherichia coli*: a global overview. Poult. Sci. 98, 1791–1804. doi: 10.3382/ps/pey539 30544256 PMC6414035

[ref54] Rothrock M. J. Hiett K. L. Guard J. Y. Jackson C. R. (2016). Antibiotic resistance patterns of major zoonotic pathogens from all-natural, antibiotic-free, pasture-raised broiler flocks in the southeastern United States. J. Environ. Qual. 45, 593–603. doi: 10.2134/jeq2015.07.0366, PMID: 27065406

[ref55] Rozynek E. Dzierzanowska-Fangrat K. Jozwiak P. Popowski J. Korsak D. Dzierzanowska D. (2005). Prevalence of potential virulence markers in polish *Campylobacter jejuni* and *Campylobacter coli* isolates obtained from hospitalized children and from chicken carcasses. J. Med. Microbiol. 54, 615–619. doi: 10.1099/jmm.0.45988-0 15947425

[ref56] Saenz Y. Zarazaga M. Lantero M. Gastanares M. J. Baquero F. Torres C. (2000). Antimicrobial resistance in *Campylobacter* strains isolated from animals, foods, and humans in Spain in 1997-1998. Antimicrob. Agents Chemother. 44, 267–271. doi: 10.1128/AAC.44.2.267-271.2000, PMID: 10639348 PMC89669

[ref57] Scuron M. D. Boesze-Battaglia K. Dlakićcy M. Shenker B. J. (2016). The cytolethal distending toxin contributes to microbial virulence and disease pathogenesis by acting as a tri-perditious toxin. Front. Cell. Infect. Microbiol. 6:168. doi: 10.3389/fcimb.2016.00168, PMID: 27995094 PMC5136569

[ref58] Shakir Z. M. Alhatami A. O. Ismail Khudhair Y. Muhsen Abdulwahab H. (2021). Antibiotic resistance profile and multiple antibiotic resistance index of *Campylobacter* species isolated from poultry. Arch. Razi Inst. 76, 1677–1686. doi: 10.22092/ari.2021.356400.1837, PMID: 35546994 PMC9083853

[ref59] Sher A. A. Ashraf M. A. Mustafa B. E. Raza M. M. (2021). Epidemiological trends of foodborne *Campylobacter* outbreaks in the United States of America, 1998–2016. Food Microbiol. 97:103751. doi: 10.1016/j.fm.2021.103751, PMID: 33653524

[ref60] Skirrow M. B. Blaser M. J. (2000). “Clinical aspects of *Campylobacter* infection” in Campylobacter. eds. Nachamkin I. Blaser M. J.. 2nd ed (Washington, D. C.: ASM Press), 69–88.

[ref61] Smid J. H. Mughini Gras L. de Boer A. G. French N. P. Havelaar A. H. Wagenaar J. A.. (2013). Practicalities of using non-local or non-recent multilocus sequence typing data for source attribution in space and time of human campylobacteriosis. PLoS One 8:e55029. doi: 10.1371/journal.pone.0055029, PMID: 23405107 PMC3566096

[ref62] Stampa E. Schipmann-Schwarze C. Hamm U. (2020). Consumer perceptions, preferences, and behavior regarding pasture-raised livestock products: a review. Food Qual. Prefer. 82:103872. doi: 10.1016/j.foodqual.2020.103872

[ref9010] Stern N. J. Wojton B. Kwiatek K. A. (1992). Differential-selective medium and dry ice-generated atmosphere for recovery of *Campylobacter jejuni*. J. Food Prot. 55, 514–517. doi: 10.4315/0362-028X-55.7.514 31071893

[ref63] Suzuki H. Yamamoto S. (2009). *Campylobacter* contamination in retail poultry meats and by-products in the world: a literature survey. J. Vet. Med. Sci. 71, 255–261. doi: 10.1292/jvms.71.255, PMID: 19346690

[ref64] Talukder K. Aslam M. Islam Z. Azmin I. Duttad D. (2008). Prevalence of virulence genes and cytolethal distending toxin production in *Campylobacter jejuni* isolates from diarrheal patients in Bangladesh. J. Clin. Microbiol. 46, 1485–1488. doi: 10.1128/JCM.01912-07, PMID: 18287317 PMC2292937

[ref65] Vacher S. Me Nard A. Bernard E. Me Graud F. (2003). PCR restriction fragment length polymorphism analysis for detection of point mutations associated with macrolide resistance in *Campylobacter* spp. Antimicrob. Agents Chemother. 47, 1125–1128. doi: 10.1128/AAC.47.3.1125-1128.2003, PMID: 12604552 PMC149329

[ref66] Van Boeckel T. P. Brower C. Gilbert M. Grenfell B. T. Levin S. A. Robinson T. P.. (2015). Global trends in antimicrobial use in food animals. Proc. Natl. Acad. Sci. U. S. A. 112, 5649–5654. doi: 10.1073/pnas.1503141112, PMID: 25792457 PMC4426470

[ref67] Varga C. Rajić A. McFall M. E. Reid-Smith R. J. McEwen S. A. (2009). Associations among antimicrobial use and antimicrobial resistance of *Salmonella* spp. isolates from 60 Alberta finishing swine farms. Foodborne Pathog. Dis. 6, 23–31. doi: 10.1089/fpd.2008.0118, PMID: 18991537

[ref68] Vetchapitak T. Misawa N. (2019). Current status of *Campylobacter* food poisoning in Japan. Food Saf. (Tokyo) 7, 61–73. doi: 10.14252/foodsafetyfscj.D-19-00001, PMID: 31998589 PMC6977775

[ref69] Wieczorek K. Denis E. Lynch O. Osek J. (2013). Molecular characterization and antibiotic resistance profiling of *Campylobacter* isolated from cattle in polish slaughterhouses. Food Microbiol. 34, 130–136. doi: 10.1016/j.fm.2012.12.003, PMID: 23498189

[ref70] Wieczorek K. Denis E. Osek J. (2015). Comparative analysis of antimicrobial resistance and genetic diversity of *Campylobacter* from broilers slaughtered in Poland. Int. J. Food Microbiol. 210, 24–32. doi: 10.1016/j.ijfoodmicro.2015.06.006 26092707

[ref71] Wieczorek K. Wołkowicz T. Osek J. (2018). Antimicrobial resistance and virulence-associated traits of *Campylobacter jejuni* isolated from poultry food chain and humans with diarrhea. Front. Microbiol. 9:1508. doi: 10.3389/fmicb.2018.01508, PMID: 30022977 PMC6039573

[ref72] Wozniak-Biel A. Bugla-Płoskonska G. Kielsznia A. Korzekwa K. Tobiasz A. Korzeniowska-Kowal A.. (2018). High prevalence of resistance to fluoroquinolones and tetracycline *Campylobacter* spp. isolated from poultry in Poland. Microb. Drug Resist. 24, 314–322. doi: 10.1089/mdr.2016.0249, PMID: 28628752 PMC5905868

[ref73] Yeh H. Cox N. A. Hinton A. Jr. Berrang M. E. Plumblee-Lawrence J. R. Thompson T. M. (2022). Prevalence and characterization of quinolone resistance in *Campylobacter* spp. isolates in chicken livers from retail stores in Georgia, USA. J. Food Prot. 85, 406–413. doi: 10.4315/JFP-21-357, PMID: 34818407

[ref74] Yeh H. Hiett K. L. Line J. E. Oakley B. B. Seal B. S. (2013). Construction, expression, purification and antigenicity of recombinant *Campylobacter jejuni* flagellar proteins. Microbiol. Res. 168, 192–198. doi: 10.1016/j.micres.2012.11.010 23312848

[ref75] Yeung R. M. W. Morris J. (2001). Consumer perception of food risk in chicken meat. Nutr. Food Sci. 31, 270–279. doi: 10.1108/00346650110409092

[ref76] Zhang T. Luo Q. Chen Y. Li T. Wen G. Zhang R.. (2016). Molecular epidemiology, virulence determinants and antimicrobial resistance of *Campylobacter* spreading in retail chicken meat in central China. Gut Pathog. 8:48. doi: 10.1186/s13099-016-0132-2, PMID: 27800028 PMC5080698

[ref77] Zhou J. Zhang M. Yang W. Fang Y. Wang G. Hou F. (2016). A seventeen-year observation of the antimicrobial susceptibility of clinical *Campylobacter jejuni* and the molecular mechanisms of erythromycin-resistant isolates in Beijing, China. Int. J. Infect. Dis. 42, 28–33. doi: 10.1016/j.ijid.2015.11.005, PMID: 26594011

[ref78] Ziprin R. L. Young C. R. Byrd J. A. Stanker L. H. Hume M. E. Gray S. A.. (2001). Role of *Campylobacter jejuni* potential virulence genes in cecal colonization. Avian Dis. 45, 549–557. doi: 10.2307/1592894, PMID: 11569726

[ref79] Ziprin R. L. Young C. R. Stanker L. H. Hume M. E. Konkel M. E. (1999). The absence of coecal colonization of chicks by a mutant of *Campylobacter jejuni* not expressing bacterial fibronectin-binding protein. Avian Dis. 43, 586–589. doi: 10.2307/1592660, PMID: 10494431

[ref80] Zirnstein G. Li Y. Swaminathan B. Angulo F. (1999). Ciprofloxacin resistance in *Campylobacter jejuni* isolates: detection of *gyrA* resistance mutations by mismatch amplification mutation assay PCR and DNA sequence analysis. J. Clin. Microbiol. 37, 3276–3280. doi: 10.1128/JCM.37.10.3276-3280.1999, PMID: 10488192 PMC85547

