# Supplementary material for: Genotypic characterization, antimicrobial susceptibility and virulence determinants of Campylobacter jejuni and Campylobacter coli isolated from pastured poultry farms
Source: Front Microbiol. 2023 Nov 9;14:1271551. doi: 10.3389/fmicb.2023.1271551 (PMC10668334; doi:10.3389/fmicb.2023.1271551)
Supplement: Supplementary file 1 [file Table_1.DOCX]

Supplementary Material

**Genotypic Characterization, Antimicrobial Susceptibility and Virulence Determinants of *Campylobacter jejuni* and *Campylobacter coli* Isolated from Pastured Poultry Farms**

[Amal Awad](https://pubmed.ncbi.nlm.nih.gov/?term=Awad%20A%5BAuthor%5D)^1^, [Hung‐Yueh Yeh](https://pubmed.ncbi.nlm.nih.gov/?term=Yeh%20H%5BAuthor%5D)^2*^, Hazem Ramadan^3^, [Michael J. Rothrock](https://pubmed.ncbi.nlm.nih.gov/?term=Rothrock%20MJ%5BAuthor%5D)^2^

**^*^ Correspondence:**

Hung-Yueh Yeh, Ph.D.

# [hungyueh.yeh@usda.gov](mailto:hungyueh.yeh@usda.gov)

Supplementary Table 1. Frequency distribution of the recovered *Campylobacter jejuni* and *C. coli* from different sources.

| Source | *C. jeju*ni (n=79)  No. (%) | *C. coli* (n=18)  No. (%) | Total (n=97)  No. (%) |
| --- | --- | --- | --- |
| Broiler feces | 35 | 1 | 36 |
| Broiler ceca | 9 | 8 | 17 |
| Whole chicken rinse | 11 | 5 | 16 |
| Broiler soil | 6 | 4 | 10 |
| Pig feces | 4 | 0 | 4 |
| Layer feces | 7 | 0 | 7 |
| Layer soil | 3 | 0 | 3 |
| Cow feces | 2 | 0 | 2 |
| Cow soil | 2 | 0 | 2 |
| P value | 0.0023* | | |

* Denotes significant (P < 0.05) associations of isolate recovery to the examined sources.
